# Natural Infection by Toscana Virus and Other Phleboviruses in Wild Sand Flies From Spain: Prevalence, Genetic Characterization and Tripartite Interactions With *Leishmania infantum* and *Wolbachia*

**DOI:** 10.1111/zph.70086

**Published:** 2026-08-20

**Authors:** Andrés Torres‐Llamas, Antonio Sampedro, Victoriano Díaz‐Sáez, Manuel Morales‐Yuste, Javier Rodríguez‐Granger, Irene Pedrosa‐Corral, José María Navarro‐Marí, Francisco Morillas‐Márquez, Joaquina Martín‐Sánchez

**Affiliations:** ^1^ Department of Parasitology, Faculty of Pharmacy University of Granada, Campus Universitario de Cartuja Granada Spain; ^2^ Service of Microbiology University Hospital Virgen de las Nieves Granada Spain; ^3^ Instituto de Investigación Biosanitaria de Granada (ibs.GRANADA) Granada Spain

**Keywords:** genetic characterization, *Leishmania infantum*, phleboviruses, prevalence, Toscana virus, *Wolbachia*

## Abstract

**Introduction:**

Toscana virus (TOSV) and *Leishmania* species are sand fly‐borne pathogens that pose a significant public health threat and an increasing risk of exposure in Europe. The objective of this study was to identify the phleboviruses circulating in an area endemic for sand fly‐borne pathogens and to estimate infection prevalence in wild sand fly populations. We also aimed to analyse co‐infections with *L. infantum* and the symbiotic bacterium *Wolbachia*.

**Methods:**

The study was conducted in southern Spain. Sand flies were collected using CDC light traps, morphologically identified and DNA/RNA was extracted individually or in pools. A generic RT‐nested PCR was used to detect phleboviruses, followed by sequencing and phylogenetic analysis. *Leishmania infantum* loads were quantified by qPCR, and *Wolbachia* was detected by PCR.

**Results:**

We found that 2/443 individual sand flies (0.45%) and 8/64 pools (12.5%) were infected with phleboviruses, with an estimated prevalence of 1.9%. We confirm the circulation of TOSV‐lineage B, together with Massilia‐like virus and Alcube virus, in three sand fly species, and report novel strains of these phleboviruses. In addition, two *Phlebotomus perniciosus* individuals were co‐infected with TOSV‐lineage B and *Wolbachia* wPrn. We also detected *L. infantum* in 27.6% (60/217) of individuals, 66.7% (40/60) of which were co‐infected with *Wolbachia*. Mean *L. infantum* loads [log‐(parasite load +1)] in specimens co‐infected with *Wolbachia* were not significantly different from those in uninfected specimens (*p* = 0.4).

**Conclusions:**

This study provides further evidence for the continued circulation of TOSV and other phleboviruses in sand fly populations in southern Spain, with an estimated prevalence higher than that reported in the same region over a decade ago. Our study provides the first evidence of co‐infection with TOSV‐lineage B and *Wolbachia* in *P. perniciosus*, as well as a high proportion of specimens co‐infected with *Wolbachia* and *L. infantum*. Understanding these co‐infection dynamics is essential to elucidate the potential role of *Wolbachia* and the impact of *Leishmania*–phlebovirus co‐infection on transmission.

## Impacts

- Novel Toscana virus, Massilia‐like virus and Alcube virus strains circulate in three sand fly species in southern Spain.
- This study provides the first evidence of TOSV‐lineage B and *Wolbachia* co‐infection in *Phlebotomus perniciosus*.
- Co‐infection between *Wolbachia* wPrn strain and *L. infantum* was detected in 66.7% of the *P. perniciosus* specimens analysed.

## 1 Introduction

Pathogens transmitted to humans by phlebotomine sand flies (Diptera, Psychodidae) remain neglected, even though infections caused by protozoa of the genus *Leishmania* (Kinetoplastida, Trypanosomatidae) and viruses from genus *Phlebovirus* (Hareavirales, Phenuiviridae) have important implications for human health. Both infections are emerging or re‐emerging in Europe (Moriconi et al. 2017; Vaselek 2021). Several environmental factors—such as climate change, increasing urbanization, population movements and the colonization of urban areas by wildlife—are driving changes in the epidemiology of sand fly‐borne infections by altering the accessible ranges of vectors, reservoirs and pathogens (Molina et al. 2012; Vadmal et al. 2023; Barroso et al. 2024; Martín‐Sánchez et al. 2024). Among the emerging challenges related to sand fly‐borne pathogens, particular attention should be given to the interactions between *Leishmania* and sand fly‐borne viruses, as well as those involving sand fly endosymbionts (Torres‐Llamas et al. 2025). These interactions could have implications for vector competence and transmission dynamics (Torres‐Llamas et al. 2025; Stejskalova et al. 2026). In Spain, *Leishmania infantum* is responsible for both canine leishmaniasis (CanL) and the three clinical forms of human leishmaniasis: visceral (VL), cutaneous (CL) and mucosal (ML), with dogs being the primary domestic reservoir and leporids the main sylvatic reservoir for the parasite (Molina et al. 2012; Martín‐Sánchez et al. 2020, 2024; Morales‐Yuste et al. 2022; Fernández‐Arévalo et al. 2024). Nevertheless, knowledge about infections caused by phleboviruses is still limited.

Phleboviruses are single‐stranded RNA viruses whose genome is segmented into three parts of different sizes, small (S), medium (M) and large (L) (Calzolari et al. 2021). Toscana virus (TOSV) is among the most pathogenic phleboviruses infecting humans and is one of the principal causes of viral meningitis in the Mediterranean region, along with enteroviruses and herpesviruses (Ayhan and Charrel 2020; Hong et al. 2024). Over the past 38 years, 1381 confirmed cases of TOSV infection have been reported (reviewed by Ayhan et al. 2025), with most cases diagnosed in Italy and Spain. Although mortality related to TOSV infections is low and appears to be associated with age and other comorbidities, a large proportion of cases present neurological manifestations such as meningitis, meningoencephalitis and other symptoms affecting the central nervous system (Ayhan et al. 2025). However, due to the lack of specific symptoms, pathognomonic features and established diagnostic protocols, it is likely that many cases remain undiagnosed or unreported (García San Miguel et al. 2021; Hong et al. 2024; Ayhan et al. 2025).

The geographical expansion of sand flies in Europe and surrounding regions poses a growing threat of exposure to TOSV, placing millions of people at risk of infection (Hong et al. 2024; Wang et al. 2025). Despite the detection of TOSV in several vertebrate animals—both serologically and, in some cases, molecularly—their role as potential reservoirs remains uncertain (Ayhan et al. 2020; Keskek Turk et al. 2024; Xhekaj et al. 2025). There are currently three genetic lineages of Toscana virus circulating in Europe, TOSV‐lineage A, B and C (Ayhan et al. 2017, 2020). While lineage A has been detected in France, Italy, Türkiye, Tunisia and Algeria, lineage B has been reported in Spain, Portugal, France, Morocco and Türkiye. In addition, lineage C has only been identified in Croatia and Greece. Notably, the co‐circulation of multiple lineages has been documented in France and Türkiye (lineages A and B) and in Croatia (lineages B and C) (reviewed by Ayhan and Charrel 2020). Recent studies suggest that TOSV‐lineage A may be more pathogenic than lineage B, due to its higher replication rate, increased virion production and greater neuroinvasive capacity (Roy et al. 2025; Thiesson et al. 2025). To date, only TOSV‐lineage B has been detected in sand flies on the Iberian Peninsula, and several viral strains have been characterized (Sanbonmatsu‐Gámez et al. 2005; Amaro et al. 2024).

The circulation of several phleboviruses other than TOSV has also been detected in Spain. Massilia virus (MASV), a species within the Sandfly fever Naples complex, was first identified in southeastern France, and has since been found in sand flies in both Portugal and Spain (Charrel et al. 2009; Amaro et al. 2015, 2021; Davó et al. 2020). Another Massilia‐like virus—tentatively named Granada virus (GRV)—was detected in sand flies in the Granada Province (southern Spain). This putative new virus shares high nucleotide homology with L and S segments of MASV (Collao et al. 2010). However, based on current International Committee on Taxonomy of Viruses (ICTV) criteria for defining new phlebovirus species, GRV should be classified within the MASV species (Davó et al. 2020; Amaro et al. 2022).

Although both phleboviruses and *Leishmania* have been found to circulate within the same geographic area, potential interactions between these sand fly‐borne pathogens remain to be elucidated (Amaro et al. 2023). The transmission of pathogens by insect vectors may be modulated not only by interactions between co‐infecting pathogens themselves, but also by their interactions with other microorganisms, including the endosymbiotic bacterium *Wolbachia pipientis* (Wong et al. 2020). *Wolbachia* can interfere with the establishment of both viral and parasitic infections in insect hosts. For instance, *Wolbachia* has been shown to protect insects against RNA viruses (Moreira et al. 2009; Pimentel et al. 2025), and when introduced into *Anopheles* mosquitoes, can induce refractoriness to *Plasmodium* infection (Bian et al. 2013). Despite these reported properties, the effects of *Wolbachia* on sand flies and the pathogens they transmit remain poorly understood (Yilmaz and Kasap 2025) and are likely influenced by both the sand fly species or lineage and the infecting *Wolbachia* strain (Stejskalova et al. 2025; Torres‐Llamas et al. 2025). Only a few experimental studies have investigated the potential interactions between *Wolbachia* (or its surface protein WSP) and *L. infantum*, and none has assessed a *Wolbachia* strain naturally associated with sand flies (da Silva Gonçalves et al. 2019; Varotto‐Boccazzi et al. 2020; Latrofa et al. 2023).

Thus, potential associations among the development of *Leishmania* parasites, phleboviruses and *Wolbachia* warrant further investigation, particularly regarding epidemiological links and the conditional capacity of these pathogens to interact in ways that may enhance transmission or virulence. To address these gaps, field surveys are needed to assess all three components within the same sand fly populations. The primary objective of this study was to identify the phleboviruses circulating in an area endemic for sand fly‐borne pathogens, such as TOSV and *L. infantum*, and to estimate the prevalence of infection in wild sand fly populations. We also focused on characterizing viral strains based on the L and S segments and establishing their phylogenetic relationships. Ultimately, we aimed to analyse tripartite co‐infections involving TOSV, *L. infantum* and the endosymbiotic bacterium *Wolbachia*.

## 2 Methods

### 2.1 Study Area

The study was conducted in Granada Province, located in southern Spain (Andalusia; centroid coordinates: 37°10′ N, 3°35′ W; Figure 1). The province spans a marked altitudinal gradient ranging from 0 to 3479 m above sea level. Five sampling sites, all of which fall within the mesomediterranean bioclimatic stage (Rivas Martínez et al. 1987), were selected in the peri‐urban area of Granada city (695–885 m a.s.l.) based on previous records of established sand fly populations. The coordinates, elevation, dominant vegetation and fauna of each site are summarized in Table 1.

**FIGURE 1:**
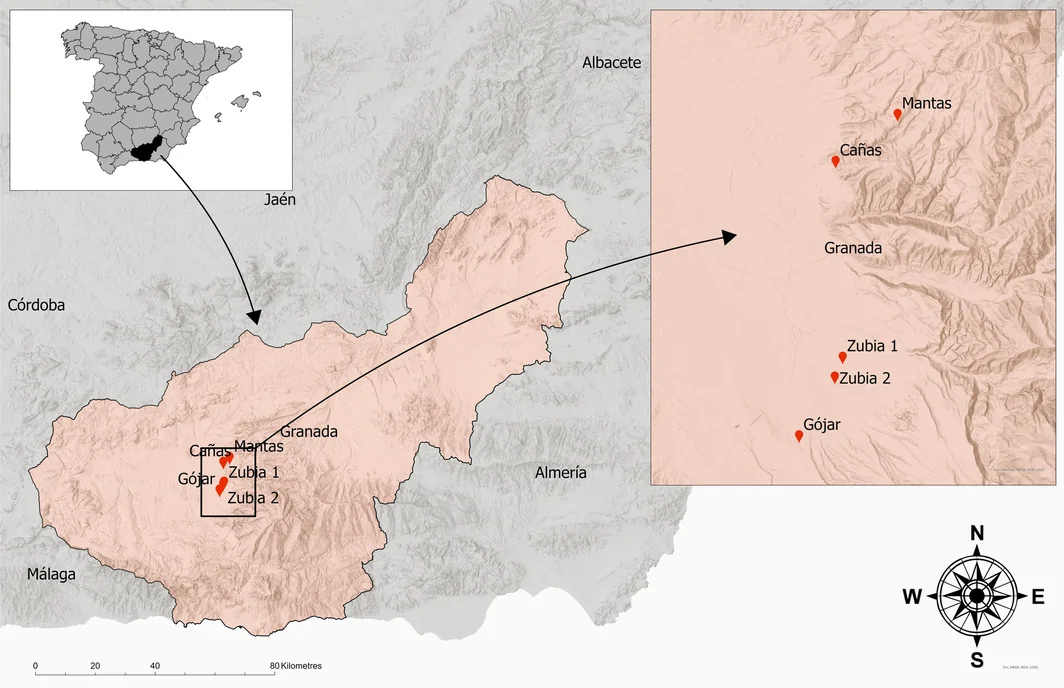
Geographical distribution of sand fly collection sites in southern Spain.

**TABLE 1:** Characteristics of the sampling sites and distribution of the sand flies collected by location, species, sex and processing method (pooled or individual).

Collection sites and features	Species	No. of specimens	No. of pools	Individuals
Mantas: At an elevation of 885 m (37°12′34.5″N 3°34′9.5″W) the predominant vegetation consists of almond, fig and olive trees and dry straw. The animals present include sheep, goats, peacocks, cats and chickens.	*P. perniciosus*	156M; 235F	23 (5F; 18M)	72M; 181F
	*P. ariasi*	36F	3 (3F)	—
	*P. papatasi*	4M; 4F	—	4M; 4F
	*P. sergenti*	3M; 2F	—	3M; 2F
	*S. minuta*	35F	1 (1F)	22F
Cañas: At an elevation of 780 m (37°11′42.4″N 3°35′34.4″W), the predominant vegetation consists of fig and olive trees, along with shrubs. The animals present include dogs, cats and rabbits.	*P. perniciosus*	81M; 53F	22 (5F; 17M)	—
	*P. ariasi*	22M; 26F	4 (2F; 2M)	—
	*P. papatasi*	5F	1 (1F)	—
	*P. sergenti*	12F	1 (1F)	—
	*S. minuta*	18M; 27F	9 (3F; 6M)	—
Gójar: At an elevation of 760 m (37°6′40.0″N 3°36′22.3″W), the predominant vegetation consists of cypresses and firewood. The animals present include dogs and cats	*P. perniciosus*	8F	—	8F
Zubia 1: At an elevation of 700 m (37°8′6.8″N 3°35′23.0″W), the predominant vegetation consists of almond and lemon trees, and dry straw. The animals present are dogs, cats, horses, mules, donkeys, goats and sheep.	*P. perniciosus*	9F	—	9F
	*S. minuta*	5F	—	5F
Zubia 2: At an elevation of 695 m (37°7′44.9″N 3°35′33.6″W), the predominant vegetation consists of almond and olive trees, and dry straw. The animals present include dogs, mules, pigs, mice, ducks, geese, chickens, turkeys, sheep, goats and rabbits.	*P. perniciosus*	22M; 19F	—	22M; 19F
	*P. papatasi*	26M; 18F	—	26M; 18F
	*P. sergenti*	12M; 17F	—	12M; 17F
	*S. minuta*	7M; 12F	—	7M; 12F
Total	Pp (*n* = 583; 66.7%)	874 (351M; 523F)	64 (21F; 43M)	443 (146M; 297F)
	Sm (*n* = 104; 11.9%)			
	Pa (*n* = 84; 9.6%)			
	Ppa (*n* = 57; 6.5%)			
	Ps (*n* = 46; 5.3%)			

Abbreviations: F, female; M, male; Pa, *P. ariasi* ; Pp, *P. perniciosus*; Ppa, *P. papatasi*; Ps, *P. sergenti*; Sm, *S. minuta* .

### 2.2 Sand Flies Collection and Species Identification

Phlebotomine sand flies were collected in 2019 and 2022 using battery‐operated CDC miniature light traps without CO_2_ bait (J. W. Hock Company, Gainesville, FL, USA). At each site, two traps were deployed for one night per month from July to September (6 trap‐nights per site and year), operating for approximately 12 h from dusk to dawn (19:00–20:00 to 07:00–08:00). Traps were placed close to animal shelters, preferentially indoors. When indoor access was not feasible, traps were deployed outdoors in the immediate peridomestic surroundings. All sampling sites were located approximately 1–3 km from the nearest residential area. After collection, the specimens were promptly transferred to the laboratory and preserved in RNAprotect Tissue Reagent (QIAGEN, Germany) at −80°C to maintain RNA integrity. For morphological identification, the genitalia were dissected on ice and mounted, whereas the remainder of the body was used for nucleic acid extraction. Identification was performed using taxonomic keys, with particular attention to the spermathecae in females and the external genitalia in males, especially the aedeagus (Sáez et al. 2018). Following identification, specimens were either processed individually or grouped into pools comprising 3–17 individuals of the same species, sex, location and date.

### 2.3 Sand Fly Nucleic Acid Extraction

Genomic DNA and RNA were extracted from the remaining sand fly tissues after morphological identification. A commercial MasterPure Complete DNA and RNA Purification Kit (cat. no. MC85200) was used according to the manufacturer's instructions. Sand flies were processed individually or in pools and then placed in a sterile 1.5 mL Eppendorf tube and kept in liquid nitrogen for a few seconds to facilitate the mechanical tissue disruption with a pestle. The nucleic acids were resuspended in 20 μL of nuclease‐free water and stored at −80°C until use.

### 2.4 Detection of Phleboviruses RNA

A generic RT‐nested PCR adapted from Sánchez‐Seco et al. (2003) was used to detect TOSV and other phleboviruses. First, reverse transcription was performed from 8 μL of RNA using SensiFAST cDNA Synthesis Kit (Cat. No. BIO‐65054, Meridian bioscience, UK), following the manufacturer's instructions. The resulting cDNA was used as a template for the first‐round PCR targeting a conserved region of the L segment with primers NPhlebo1+/NPhlebo1–. The first‐round product was then used as a template for two parallel nested reactions: (i) a nested‐PCR with primers NPhlebo2+/NPhlebo2–, capable of detecting a broad range of phlebovirus species; and (ii) a TOSV‐specific nested‐PCR in which NPhlebo2– was replaced by the TOSV‐specific antisense primer ATos2−. Amplicons from the generic nested PCR were sequenced using the same primers (NPhlebo2+/NPhlebo2−). Additionally, the S segment was amplified using primers TosN123/TosN829 in the first PCR and TosN234/TosN794 in the nested‐PCR, with the latter also used for sequencing. Primer sequences and amplification conditions are provided in Supporting Information. RNA extracted from a TOSV strain isolated elsewhere was used as a positive control (Sánchez‐Seco et al. 2003). A negative control containing all reagents and nuclease‐free water was included in each PCR run.

### 2.5 Sequencing and Phylogenetic Analysis

Amplified PCR products were eluted from agarose gel using Real Clean Spin Kit (Zymoclean Gel DNA Recovery Kit, cat. no. D4007), according to the manufacturer's protocol. Direct cycle Sanger sequencing of the PCR‐amplified products was performed bidirectionally using an ABI 3130XL automated sequencer. The resulting sequences were visualized and manually curated with Chromas version 2.6.6, then aligned using ClustalW as implemented in MEGA11. The best nucleotide substitution model was selected using jModelTest version 2.1.10 and MEGA11. Phylogenetic relationships among phlebovirus consensus sequences from both L and S segments, as well as additional sequences available in GenBank, were inferred using MEGA11. Genetic distances were calculated using the Kimura 2‐parameter (K2P) model. Three phylogenetic methods were applied: Neighbour‐Joining (NJ), Maximum Likelihood (ML) and Maximum Parsimony (MP). The NJ and ML trees were inferred under the best‐fitting substitution model for both segments (K2P+G), whereas the MP tree was inferred using the max‐mini branch‐and‐bound search method. Model selection was based on the lowest Akaike Information Criterion (AIC) score. All positions containing gaps or missing data were excluded from the analyses using the complete deletion option. The robustness of internal branches was assessed by bootstrap analysis with 1000 replicates, and values below 50% were hidden. Uukuniemi virus sequences for both L (Accession: M33551) and S (Accession: KM114249) segments were used as an outgroup.

### 2.6 Determination of *Leishmania infantum* Loads by qPCR

GRANALEISH Multiplex qPCR (University of Granada, Spain, Trademark number 3667362/5) (Gijón‐Robles et al. 2018) was used for identification of *L. infantum* and quantification of the parasite load. Primers and the TaqMan probe were provided by the manufacturer. The thermal cycling profile used was as follows: an initial denaturation at 95°C for 10 min, followed by 40 cycles of 30 s at 95°C and 60 s at 60°C. A standard curve was constructed from serial dilutions of DNA extracted from cultured *L. infantum* strain MHOM/ES/14/DP581 representing concentrations equivalent to 1000–0.009 promastigotes/μL. The standard curve showed a coefficient of determination (*R* ^2^) of 0.975 and a PCR efficiency of 99.5%. Parasite numbers in each qPCR reaction were calculated by interpolating the Ct values against the standard curve. DNA extracted from the cultured *L. infantum* reference strain MHOM/ES/14/DP581 was used as a positive control. A no‐template control containing all reagents and nuclease‐free water was included in each PCR run. Additionally, negative extraction controls were used to monitor potential cross‐contamination.

### 2.7 Detection of *Wolbachia* DNA and Sequencing

The presence of *Wolbachia* DNA in sand flies was assessed using conventional PCR. Primers 81F (5′‐TGG TCC AAT AAG TGA TGA AGA AAC‐3′) and 691R: (5′‐AAA AAT TAA ACG CTA CTC CA‐3′) were used to amplify a fragment of 590–632 base pairs (bp) of the *Wolbachia wsp* gene. The variability in amplicon size reflects natural sequence length polymorphism in the *wsp* gene among *Wolbachia* strains (Zhou et al. 1998). The reagent quantities and PCR final volume used in this study follow the protocol described by Torres‐Llamas et al. (2025). The amplification conditions were: 2 min denaturation at 94°C followed by 35 cycles of denaturation at 94°C for 30 s, annealing at 55°C for 30 s and extension at 72°C for 1 min, with a final extension at 72°C for 10 min. Sand fly specimens that tested positive or negative for *Wolbachia* in a previous study (Torres‐Llamas et al. 2025) were used as positive and negative controls, respectively. Positive specimens were sequenced to determine the *Wolbachia* strain.

### 2.8 Data Analysis

Sand flies were processed and phleboviruses detected using two approaches: individual sampling and pooled sampling. For the individual samples (Method 1), the number of positive specimens was recorded, and the observed prevalence was calculated as the proportion of infected individuals out of the total tested. Infection prevalence was also estimated from pooled samples of different sizes (Method 2). The infection rate in pooled samples was obtained using Maximum Likelihood Estimation (MLE). This approach defines the infection rate as the proportion of infected individuals that maximizes the likelihood of observing the number of positive and negative pools of the recorded sizes. The method assumes a binomial distribution of infected individuals within each pool and identifies the prevalence value that best explains the observed pooling results (Khongpetch et al. 2025). These analyses were performed in R v. 4.5.2.

For the analysis of interactions within the vector only individualized female *P. perniciosus* sand flies were used (*n* = 217). To investigate potential associations between *L. infantum* and *Wolbachia* infections, a logistic regression model was fitted with *L. infantum* infection status (presence/absence) as the dependent variable, and *Wolbachia* presence and sampling site as independent variables. Sampling site was included as a covariate to account for potential geographic confounding, as prevalence of *L. infantum* differed among sites. Given the uneven distribution of specimens across sampling sites, they were grouped for the regression analysis into Mantas (the main site, *n* = 181) and all remaining sites combined (*n* = 36) to obtain more stable model estimates and avoid complete separation. The effect of *Wolbachia* presence on *L. infantum* parasite load was assessed by logistic regression using a log‐transformation of parasite load [log (parasite load +1)] for variable normalization. All tests were conducted using IBM SPSS Statistics v. 21.0 for Windows (IBM Corp., Armonk, NY, USA).

## 3 Results

### 3.1 Phleboviruses Detection in Sand Flies

The presence of phleboviruses was assessed in 874 sand fly specimens collected from five locations in southern Spain, 351 males (40.2%) and 523 females (59.8%), belonging to two genera—*Phlebotomus* and *Sergentomyia*—and five species (Table 1). A total of 443 specimens were individually analysed (Table 1), of which 205 were captured in 2019 and 238 in 2022. *Phlebovirus* infection was detected in only two individually analysed female *P. perniciosus* specimens (2/443), yielding an observed prevalence of 0.45% (95% CI: 0.05%–1.62%). Both specimens were collected at the same sampling site (Mantas), albeit in different years—one in 2019 and the other in 2022. Only one of the two genomic segments analysed per specimen was successfully amplified: the L segment in one case and the S segment in the other (Table 2).

**TABLE 2:** Sand fly individuals and pools positive for phleboviruses and co‐detection of *L. infantum* and *Wolbachia*.

ID	Location	Pool size	Sand fly species	Sex	*Phlebovirus* detection (L/S)	GenBank nucleotide homology (countries)	*L. infantum* detection	*Wolbachia* detection
I 128	Mantas	−	*P. perniciosus*	Female	TOSV (S)	96%–98% (Spain, Portugal, France, Austria, Türkiye)	−	+
I 183	Mantas	−	*P. perniciosus*	Female	TOSV (L)	93%–98% (Spain, France, Morocco, Türkiye, Tunisia)	−	+
Pool 10	Cañas	9	*P. perniciosus*	Female	TOSV (L)	95%–99% (Spain, France, Morocco, Türkiye, Tunisia)	+	+
Pool 13	Mantas	3	*P. perniciosus*	Male	MASV (L)	95%–98% (Spain, Portugal, France)	NA	−
Pool 19	Mantas	3	*P. perniciosus*	Male	ACBV (L)	94%–95% (Spain and Portugal)	NA	+
					MASV (S)	90%–98% (Spain, Portugal, France)		
Pool 24	Mantas	3	*P. perniciosus*	Male	TOSV (L)	95%–99% (Spain, France, Morocco, Türkiye, Tunisia)	NA	−
					TOSV (S)	97%–99% (Spain, Portugal, France, Austria, Türkiye)		
Pool 26	Mantas	3	*P. perniciosus*	Male	MASV (S)	96%–98% (Spain, Portugal, France)	NA	−
Pool 44	Cañas	15	*P. perniciosus*	Male	TOSV (L)	95%–98% (Spain, France, Tunisia, Türkiye)	NA	+
Pool 54	Cañas	11	*P. ariasi*	Male	TOSV (L)	95%–99% (Spain, France, Morocco, Türkiye, Tunisia)	NA	+
					TOSV (S)	97%–99% (Spain, Portugal, France, Austria, Türkiye)		
Pool 64	Cañas	3	*S. minuta*	Male	TOSV (L)	95%–99% (Spain, France, Morocco, Türkiye, Tunisia)	NA	−

*Note:* Parentheses indicate amplified RNA segment.

Abbreviation: NA, not applicable.

The remaining 431 specimens, all captured in 2019, were grouped in 64 pools containing between 3 and 17 individuals each. Of these 64 pools, 21 were composed of females and 43 of males (Table 1). In total, eight pools (12.5%) tested positive for phlebovirus infection. Among the positive pools, six were from *P. perniciosus*, one from *P. ariasi* , and one from *S. minuta* (Table 2). The MLE‐based infection rate for all sand fly species was 1.9% (95% CI: 0.9%–3.6%). Three out of eight positive pools showed amplification of both genomic segments, four pools were positive exclusively for the L segment, and one pool for the S segment (Table 2).

### 3.2 Identification and Characterization of Phleboviruses Species

Following amplification of either the L or S segment, or both, Sanger sequencing enabled the identification of three phleboviruses circulating in the study area: Toscana virus (TOSV), Massilia‐like virus (MASV) and Alcube virus (ACBV).

#### 3.2.1 Toscana Virus (TOSV)

Toscana virus was detected in two *P. perniciosus* individuals, three *P. perniciosus* pools, one *Phlebotomus ariasi* pool and one *Sergentomyia minuta* pool, in one or both genomic segments (Table 2). Sequences obtained from pools were identical within each genomic segment, whereas both individual sand flies–derived sequences showed a small number of nucleotide polymorphisms for each genomic segment. BLAST analyses revealed 93%–99% nucleotide homology for both genomic segments with TOSV strains previously reported in several countries (Table 2). When all sequences were aligned, including those previously reported from the same study area (Granada) and other regions of Spain, 14 to 38 polymorphic sites were identified depending on the genomic segment analysed. Nucleotide polymorphisms among the sequences obtained in this study were all synonymous at the amino acid level. Compared with reference sequences from GenBank, a single amino acid substitution was identified in the L segment. This substitution was not shared with previously reported sequences from Granada but was consistent with those reported from other regions of Spain (Table 3). Genetic distances between TOSV‐lineage A and B ranged from 0.005 to 0.072 for the L segment and TOSV‐lineage A, B and C ranged from 0.013 to 0.195 for the S segment, with the greatest divergence observed relative to lineage C strains.

**TABLE 3:** Genetic characterization of phleboviruses circulating in the study area compared with Spanish reference strains from different years.

Phlebovirus	ID samples	Nucleotide polymorphisms	Amino acid sequence (residue/change)	Strain reference (Spain)
TOSV (S)	I128	38	—	Granada (2004, 2017), Córdoba (2015, 2017, 2018, 2019)
	Pools 24 and 54	33	—	Granada (2004, 2017), Córdoba (2015, 2017, 2018, 2019)
TOSV (L)	I183, Pools 10, 24, 44, 54 and 64	14	30/threonine to alanine^1^	Granada (2004, 2017), Córdoba (2015, 2017^1^, 2018, 2019), Madrid (2012)
MASV (S)	Pool 19 and 26	21	162/aspartic acid to asparagine	Granada (2004)
MASV (L)	Pool 13	25	9/alanine to serine^2^, 36/isoleucine to valine^3^	Granada (2004)^2^, Barcelona (2001–2004), Cabrera Island (2012)^3^
ACBV (L)	Pool 19	9	—	Formentera Island (2012)

*Note:* Nucleotide polymorphisms represent the total number of variable positions identified in a multiple sequence alignment including our sequences and all the reference strains listed. Superscript numbers (^1–3^) indicate the reference strain(s) relative to which each amino acid change was detected in our sequences. —: no amino‐acid changes detected.

Abbreviations: ACBV, Alcube virus; I, individually processed specimen; L, large genomic segment; MASV, Massilia virus; S, small genomic segment; TOSV, Toscana virus.

#### 3.2.2 Massilia‐Like Virus (MASV)

Massilia‐like virus was detected in three *P. perniciosus* pools through the amplification of either the S or L segment. Sequence homology revealed 90%–98% nucleotide homology with previously reported MASV sequences from Spain, France and Portugal (Table 2). Twenty‐one polymorphisms were detected when the S‐segment sequences were aligned with one previously reported in Granada in 2004 (then referred to as Granada virus), and only a single amino‐acid change was observed (Table 3). Similarly, 25 polymorphic sites were identified when the L segment sequence obtained in this study was aligned with those previously reported from Granada, Barcelona and Cabrera Island (Balearic Archipelago, Spain). These polymorphisms resulted in two amino‐acid changes (Table 3). Genetic distances relative to GenBank sequences ranged from 0.024 to 0.037 for the L segment and from 0.017 to 0.047 for the S segment.

#### 3.2.3 Alcube Virus (ACBV)

Alcube virus sequence was detected in a *P. perniciosus* pool (L segment), showing 94%–95% nucleotide homology with strains from Portugal and Formentera Island (Spain), (Table 2). When compared with the Formentera strain, nine polymorphisms were identified in the amplified segment (Table 3). All observed polymorphisms were synonymous at the amino‐acid level, and genetic distances ranged from 0.047 to 0.053 when compared with the Formentera Island and Portugal strains, respectively.

### 3.3 Phylogenetic Analysis of Phleboviruses

Eight new sequences have been deposited in GenBank under the following accessions: PX982977‐PX982984. Both the L and S segments of the phleboviruses detected in this study, along with other phlebovirus strains and species available in GenBank, were used to construct a phylogenetic tree. The three methods employed to infer phylogenetic relationships—Neighbour‐Joining (NJ), Maximum Likelihood (ML) and Maximum Parsimony (MP)—produced similar topologies. Neighbour‐Joining trees for both S (Figure 2a) and L (Figure 2b) segments are shown. All sequences corresponding to TOSV were clustered within lineage B, which belongs to the Sandfly fever Naples complex. Similarly, the MASV sequences were clustered with other reported sequences of this virus detected in several countries, such as France and Portugal, which belong to the same complex. In contrast, the sequence belonging to Alcube virus was clustered with other sequences reported in Spain and Portugal, which belong to the Salehabad complex.

**FIGURE 2:**
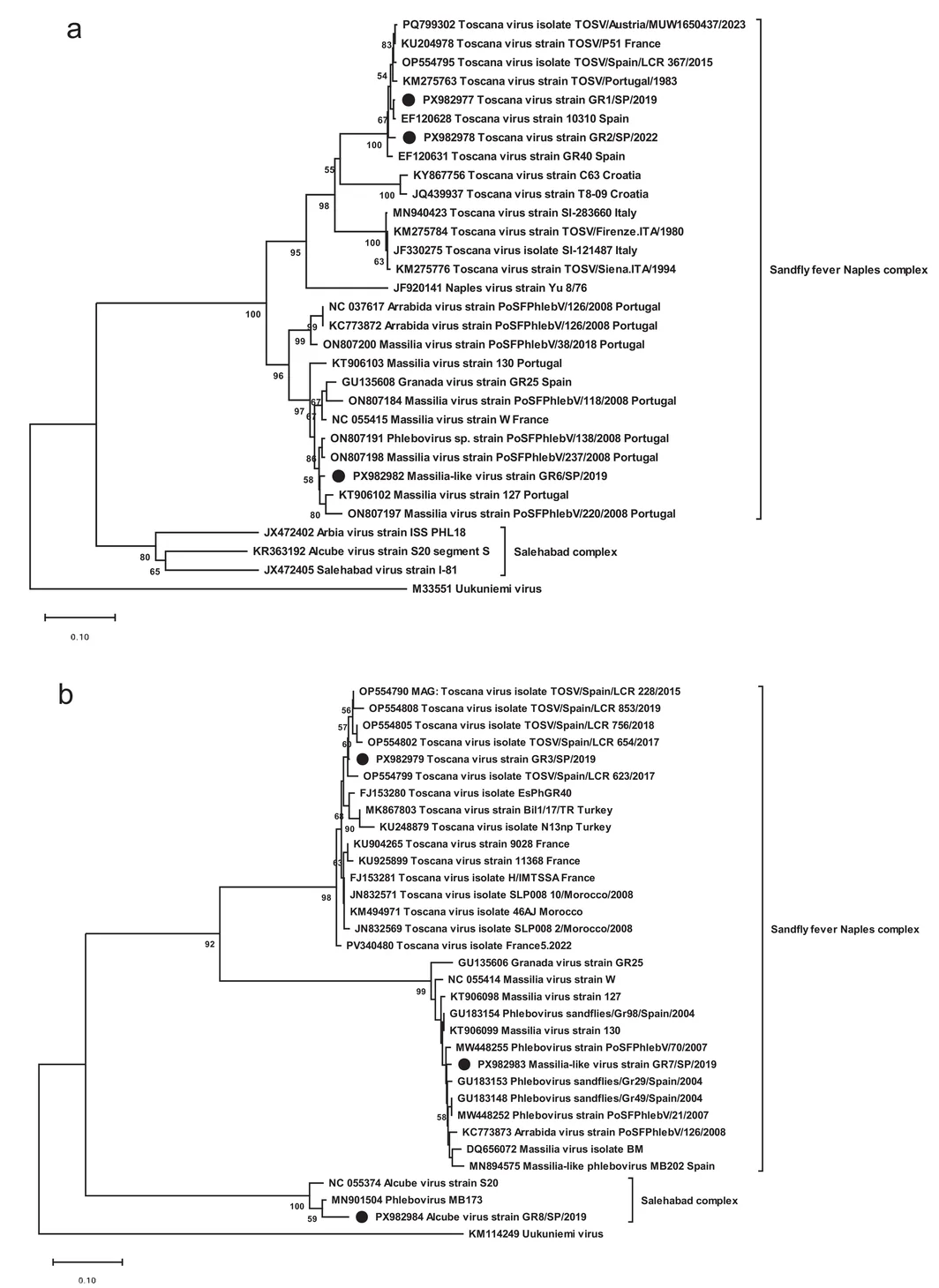
Neighbour‐Joining trees illustrating the phylogenetic relationships of the phlebovirus strains detected in this study (indicated with a circle) for the S segment (a) and the L segment (b), and their distribution within the different phlebovirus complexes.

### 3.4 Multi‐Microbe Co‐Infections in *Phlebotomus perniciosus*

Potential interactions among *Leishmania*, TOSV and *Wolbachia* were analysed in 217 individual female specimens of the species *P. perniciosus*. The prevalence of infection was 27.6% (60/217) for *L*. *infantum*, 0.9% (2/217) for TOSV and 64.1% (139/217) for *Wolbachia* wPrn strain, as determined by sequencing. Co‐infection with *L*. *infantum* and *Wolbachia* was detected in 40 specimens, representing 66.7% of *L. infantum*‐infected individuals. Logistic regression revealed no significant association between *L. infantum* and *Wolbachia* infection after adjusting for sampling site (OR = 1.15, 95% CI: 0.61–2.18, *p* = 0.663). Sampling site was the only significant predictor of *L. infantum* infection, with lower odds of infection at sites other than Mantas (OR = 0.28, 95% CI: 0.09–0.83, *p* = 0.022). No co‐infection between *L. infantum* and TOSV was observed. Both TOSV‐positive specimens were also positive for *Wolbachia*; however, given the extremely small number of phlebovirus‐positive sand flies (*n* = 2), these observations are purely descriptive and do not allow an assessment of a potential association between TOSV and *Wolbachia*. Among the 60 *L. infantum*‐infected sand flies, parasite load ranged from 0.01 to 675 parasites per specimen. *Wolbachia*‐uninfected females had a mean [log‐(parasite load +1)] of 0.826 (95% CI: 0.405–1.246), whereas *Wolbachia*‐infected females had a mean value of 0.650 (95% CI: 0.429–0.870). Logistic regression revealed no significant association between the two variables (OR = 0.743, 95% CI: 0.373–1.482, *p* = 0.4).

## 4 Discussion

Although TOSV is an emerging pathogen in the Mediterranean Basin with the potential to spread to other parts of Europe, it continues to be neglected (Laroche et al. 2024). Monitoring sand fly‐borne pathogens is essential for implementing control measures, which remain the most reliable strategy for preventing vector‐borne diseases (Wilson et al. 2020). In this study, TOSV and other phleboviruses were detected circulating in three sand fly species. The higher estimated prevalence obtained through pooled sampling may suggest that this method is more effective in detecting low‐level infections. Entomological surveys have been conducted to assess phlebovirus infection in sand fly populations across countries neighbouring Spain, including Italy, Portugal, France and Morocco (Charrel et al. 2007; Es‐Sette et al. 2012, 2015; Calzolari et al. 2021; Amaro et al. 2023, 2024). Reported phlebovirus infection rates vary by region, country and sampling year. In Spain, Sanbonmatsu‐Gámez et al. (2005) reported a pool‐based prevalence of 0.05% in the Granada Province (southern Spain), which is considerably lower than the MLE‐based prevalence of 1.9% estimated in this study. In contrast, Remoli et al. (2016) detected phleboviruses in 6 out of 50 pools analysed (12.0%) in the Community of Madrid whereas Collao et al. (2010) identified 12 positive pools among the 103 tested (11.6%) from Granada and Ibiza Island (Spain), including sequences corresponding to MASV. Despite the similar proportion of positive pools reported in these studies and in the present study (8/64; 12.5%), direct comparisons should be interpreted with caution, as they are affected by pool size and the proportion of positive pools does not directly reflect individual infection prevalence.

Overall, pooled sampling proves useful for surveillance in low‐prevalence settings, whereas individual testing remains essential for confirmation and co‐infection studies (Huemer et al. 2017). Since most studies rely on pooled samples, extrapolating our findings from individually analysed specimens remains challenging. Nevertheless, a study conducted in France individually screened 427 specimens and identified five phlebovirus‐infected individuals, although none tested positive for TOSV (Peyrefitte et al. 2013). The resulting infection rate was 1.2% (5/427), compared to the 0.45% (2/443) observed in our study. These findings underscore the need for further efforts to assess individual infection status, which is critical for elucidating co‐infection dynamics at the specimen level. In the present study, TOSV was detected in one individual specimen in each sampling year (2019: 1/205 individuals; 2022: 1/238 individuals). However, because sampling was limited to two non‐consecutive years, these data do not allow any inference of temporal trends or the stability of phlebovirus prevalence in the study area. We also compared these results with those reported by Sanbonmatsu‐Gámez et al. (2005), who sampled the same site (Mantas, Table 1) more than a decade earlier. This comparison revealed an increase in prevalence over time, as well as several silent polymorphisms among the sequences. The sequences detected for each genomic segment in the pools were identical to each other for each phlebovirus, but showed several polymorphic differences compared with individual specimens. In addition, we report new sequences of MASV and ACBV that differ from those previously described by other authors in earlier years (Collao et al. 2010; Amaro et al. 2015; Davó et al. 2020), although all of them were also synonymous at the amino acid level. This finding underscores the importance of molecular surveillance, as mutations in RNA viruses may lead to increased pathogenicity (Bakhshandeh et al. 2021).

*Phlebotomus perniciosus* and *Phlebotomus perfiliewi* are thought to be the main vectors of TOSV in the Mediterranean Basin (Hong et al. 2024). Moreover, viral RNA has been detected in other species such as *Sergentomyia minuta*, *Phlebotomus longicuspis* and *Phlebotomus sergenti*, suggesting their role as potential vectors (Charrel et al. 2006; Es‐Sette et al. 2014, 2015). Recent findings indicate that sand fly susceptibility to TOSV infection is influenced by both viral lineage and sand fly species. Notably, *Phlebotomus tobbi* and, to a lesser extent, *P. sergenti* were found to be susceptible to experimental infection with TOSV‐lineage B and therefore capable of transmitting the virus (Jancarova et al. 2025). Here, we report the natural infection of TOSV‐lineage B in *P. perniciosus* (6 pools +2 individuals), *P. ariasi* (1 pool) and *S. minuta* (1 pool). These species of sand flies are thought to be either vectors of TOSV or contributors to maintaining the virus and its transmission (Ayhan et al. 2020; Rocha et al. 2025). To our knowledge, this is the first report of *P. ariasi* being naturally infected by TOSV and the second record of *S. minuta* infected by the same virus—representing the first time this species has been found infected in Spain. Phylogenetic analyses show that the circulating strains of TOSV based on L and S segments in southern Spain belong to lineage B, which is consistent with previous reports from the same area (Sanbonmatsu‐Gámez et al. 2005).

Although considered a neglected pathogen, TOSV continues to be the main cause of meningitis during the summer months and its seroprevalence in the Mediterranean region highlights the need to strengthen epidemiological surveillance (Martinez et al. 2022; Rocha et al. 2025). Furthermore, while the animals that may act as potential reservoirs for TOSV remain unknown, several seroepidemiological studies have detected neutralizing antibodies against the virus in various mammalian species (Ayhan et al. 2020; Xhekaj et al. 2025). In southern Spain, anti‐TOSV antibodies were detected in domestic animals (dogs, cats, sheep, goats, cows, horses and pigs), with a substantial proportion (36.2%) testing positive (Navarro‐Marí et al. 2011). In that same study, TOSV RNA was also detected in a goat from a locality considered to be hyperendemic for the virus. The widespread distribution of TOSV vectors on the Iberian Peninsula, together with the presence of these animals in an area where livestock farming is a major economic activity, highlights the need for further epidemiological studies involving both humans and animals to better elucidate this issue.

Massilia‐like virus has also been detected in humans from southern Spain through IgG detection (Collao et al. 2010; Navarro‐Marí et al. 2013), although its impact on human health remains unclear. Here, we detect MASV in three sand fly pools (Table 2). However, the L segment of one of them exhibited 94%–95% homology with the ACBV, which belongs to the Salehabad complex (Amaro et al. 2015). This species was first identified in the Arrábida region of Portugal and has also been detected in a pool of *P. perniciosus* from Formentera Island (Balearic Archipelago, Spain) (Amaro et al. 2015; Davó et al. 2020). Interspecies phleboviruses resulting from genetic reassortments have been previously reported, which may lead to increased pathogenicity or virulence (Harris et al. 2025). Because these sequences were obtained from a pool, it is not possible to determine whether the L and S segments originated from a single infected specimen—potentially having undergone genetic reassortment—or from two distinct sand fly individuals infected within the same pool. Confirmation of reassortment requires sequencing of individually processed specimens and should be a priority for future studies. In most positive samples of this study, only one of the two genomic segments (L or S) was successfully amplified. This may reflect a low viral load in naturally infected sand flies and the variable performance of generic degenerate primers across phlebovirus species and genomic segments. Comparative assessments have shown that these primer sets, including the nested primers used here, yield weak or absent amplification for certain phlebovirus strains owing to primer–template mismatches (Polat et al. 2024). In addition, RNA secondary structures can reduce the accessibility of the target molecule and therefore the sensitivity of RT‐PCR assays (Weidmann et al. 2008). While pooled sampling may increase the sensitivity of detecting infected specimens, analysis of individual sand flies remains essential for confirming reassortment events. Similarly, further research is warranted to assess the impact of MASV and ACBV on human health.

Interactions between phleboviruses and *Leishmania*, as well as with the endosymbiont *Wolbachia*, remain unclear. The circulation of TOSV and *Leishmania* in the same area has previously been documented, and in some cases, both pathogens have been detected within the same pool of sand flies (Ergunay et al. 2014; Calzolari et al. 2021). Indeed, the co‐circulation of MASV and *L. infantum* has also been reported (Faucher et al. 2014). Here, we found no co‐infection between *Phlebovirus* and *Leishmania* in any of the female *P. perniciosus* individuals analysed. The experimental evidence on *Phlebovirus*–*Leishmania* interactions is still limited. In vertebrate hosts and cell cultures, phleboviral co‐infection generally enhances *Leishmania* infection (Rossi et al. 2017; Rath et al. 2019). In contrast, the only experimental study conducted in sand flies found competitive interactions, with *L. infantum* reducing TOSV infection rates in *P. tobbi*, which may explain why co‐infected sand flies are rarely detected in the field (Stejskalova et al. 2026). These interactions may involve the vector's immune responses and midgut microbiome. Accordingly, our finding should be interpreted with caution, as the absence of TOSV–*Leishmania* co‐infection is compatible with a potential negative interaction; however, this interpretation is limited by the very small number of TOSV‐positive specimens (*n* = 2).

The relatively high prevalence of *L. infantum* infection detected in *P. perniciosus* in this study (27.6%) underscores the ongoing public health relevance of leishmaniasis in Spain. Notably, this figure is consistent with the *L. infantum* infection rate of 24.0% recently reported for *P. perniciosus* collected in and around the dwellings of autochthonous human leishmaniasis cases in areas of Granada and Jaén provinces, where incidence rates of 15.1 and 8.3 human cases/100,000 inhabitants/year were reported, respectively (Martín‐Sánchez et al. 2026). Furthermore, the public health importance of *L. infantum* has been highlighted by other outbreaks. The one that occurred in the Madrid region (2009–2012) remains the largest documented outbreak of human leishmaniasis in Western Europe, with an incidence of 22.2 cases/100,000 inhabitants/year (Arce et al. 2013). Another outbreak was reported in Córdoba (southern Spain) between 2017 and 2021, resulting in a cumulative incidence of 10.3 cases/100,000 inhabitants (Barbero‐Moyano et al. 2025). In all instances, *P. perniciosus* was identified as the main vector. These findings illustrate the continuing capacity of *L. infantum* to cause significant human disease in Spain and underscore the importance of detecting high infection rates in vector populations as part of entomological surveillance.

The simultaneous presence of *Wolbachia* and *Leishmania* within the same sand fly vector has been reported in neotropical phlebotomine species (Rosário et al. 2022). In this study, *Wolbachia*–*Leishmania* co‐infection was detected in 40 *P. perniciosus* specimens, representing 66.7% of *L. infantum*‐infected individuals of this sand fly species. However, this high proportion may simply reflect the high background prevalence of *Wolbachia* rather than a specific biological interaction between the two microorganisms. This high *Wolbachia* prevalence is consistent with previous reports for *P. perniciosus* in the same region, where infection rates above 60% have been documented (Torres‐Llamas et al. 2025). The *Wolbachia* strain detected in *P. perniciosus* in this study corresponded to wPrn, with 100% homology to the strain previously identified in this species in Spain, Italy and Morocco (Torres‐Llamas et al. 2025). When *L. infantum* parasite loads were compared, no significant difference was observed between *Wolbachia*‐infected and *Wolbachia*‐uninfected females (*p* = 0.4). Although different *Wolbachia* strains have been proposed to interact with *Leishmania* spp., either enhancing or inhibiting parasite development (Torres‐Llamas et al. 2025), the role of wPrn in *L. infantum* infection remains unclear. This question can only be addressed through broader epidemiological and experimental studies, ideally incorporating quantification of *Wolbachia* density. Furthermore, the two TOSV‐infected individuals also harboured *Wolbachia*, showing that co‐infection with phleboviruses and wPrn can occur in *P. perniciosus*.

Although *Wolbachia* can inhibit the replication of several RNA viruses in other insects, such antiviral effects likely depend on the strain and the host (Teixeira et al. 2008; Moreira et al. 2009). Moreover, given the limited number of TOSV‐positive individuals, no mechanistic conclusions can be drawn regarding the interaction between wPrn and TOSV. Further studies including larger numbers of phlebovirus‐positive specimens, ideally combined with *Wolbachia* quantification, are required to assess whether wPrn modulates phlebovirus infection in *P. perniciosus*. Finally, no triple infection was detected in individual *P. perniciosus* specimens, although it was observed in one of the analysed pools (Table 2). However, it could not be confirmed whether the pathogens were present in the same individual.

Some limitations of this study should be noted, such as the small number of specimens analysed, the absence of infectivity assays, and the lack of data on vertebrate hosts, which can be mainly addressed through One Health approaches.

## 5 Conclusions

We confirm the circulation of TOSV‐lineage B, along with other phleboviruses, in southern Spain. While the estimated prevalence has increased when compared with data from more than a decade ago in that region, further longitudinal studies are needed to monitor the circulation of TOSV and estimate its prevalence over time. Despite differences in infection prevalence between individually analysed specimens and pools, both approaches provide valuable insights in epidemiological contexts. We also report new strains of TOSV‐lineage B, MASV and ACBV. Although we detected several polymorphisms across the three viral species, their amino acid sequences remain relatively conserved. Our study provides the first evidence of co‐infection with TOSV‐lineage B and *Wolbachia* in *P. perniciosus*, as well as a high percentage of specimens co‐infected with *Wolbachia* and *L. infantum*. Understanding these co‐infection dynamics is essential to elucidate the potential role *Wolbachia* may play in modulating other pathogens in sand flies, as well as for assessing the potential impact of *Leishmania*–*Phlebovirus* co‐infection on transmission to humans and other animals.

## Author Contributions

**Andrés Torres‐Llamas:** writing – review and editing, writing – original draft, visualization, validation, methodology, formal analysis, data curation, conceptualization. **Antonio Sampedro:** writing – review and editing, visualization, validation, resources, investigation, formal analysis. **Victoriano Díaz‐Sáez:** visualization, validation, resources, methodology, investigation, formal analysis. **Manuel Morales‐Yuste:** writing – review and editing, visualization, validation, software, investigation, data curation. **Javier Rodríguez‐Granger:** visualization, validation, resources, investigation, formal analysis, data curation. **Irene Pedrosa‐Corral:** visualization, validation, resources, investigation, formal analysis. **José María Navarro‐Marí:** writing – review and editing, visualization, validation, resources, investigation, formal analysis. **Francisco Morillas‐Márquez:** writing – review and editing, visualization, validation, methodology, investigation, data curation. **Joaquina Martín‐Sánchez:** writing – review and editing, writing – original draft, visualization, validation, supervision, project administration, funding acquisition, formal analysis, data curation, conceptualization.

## Funding

This study was funded by the Spanish Ministry of Science, Innovation and Universities, MICIU/AEI/10.13039/501100011033 and FEDER, EU through project PID2022‐142230NB‐I00. Andrés Torres‐Llamas was financed by a predoctoral contract FPI of the Spanish Ministry of Science, Innovation and Universities and FSE+ (grant number PREP2022‐000570). Funding for open access charge: Universidad de Granada/CBUA.

## Ethics Statement

This study did not involve humans or animals and therefore did not require approval by an ethics committee.

## Conflicts of Interest

The authors declare no conflicts of interest.

## Supporting information


**Supporting Information: S1.** Primers used in the L segment.

## Data Availability

Data is provided within the manuscript or Supporting Information. Eight sequences have been deposited in GenBank under the following accessions: PX982977–PX982984.

## References

[zph70086-bib-0001] Amaro, F. , A. Vilares , S. Martins , et al. 2023. “Co‐Circulation of *Leishmania* Parasites and Phleboviruses in a Population of Sand Flies Collected in the South of Portugal.” Tropical Medicine and Infectious Disease 9, no. 1: 3. 10.3390/tropicalmed9010003. PMC10821132 38276633

[zph70086-bib-0002] Amaro, F. , L. Zé‐Zé , M. J. Alves , et al. 2015. “Co‐Circulation of a Novel Phlebovirus and Massilia Virus in Sandflies, Portugal.” Virology Journal 12: 174. 10.1186/s12985-015-0407-0. PMC4619550 26497645

[zph70086-bib-0003] Amaro, F. , L. Zé‐Zé , and M. J. Alves . 2022. “Sandfly‐Borne Phleboviruses in Portugal: Four and Still Counting.” Viruses 14, no. 8: 1768. 10.3390/v14081768. PMC9413822 36016390

[zph70086-bib-0004] Amaro, F. , L. Zé‐Zé , J. Lourenço , M. Giovanetti , S. C. Becker , and M. J. Alves . 2021. “Phylogenetic Analysis of *Massilia phlebovirus* in Portugal.” Viruses 13, no. 7: 1412. 10.3390/v13071412. PMC8310352 34372617

[zph70086-bib-0005] Amaro, F. , L. Zé‐Zé , H. C. Osório , et al. 2024. “Toscana Virus in Wild‐Caught Sand Flies in Portugal, Findings From the National Vector Surveillance Network, 2023.” Pathogens 13, no. 10: 905. 10.3390/pathogens13100905. PMC11510618 39452776

[zph70086-bib-0006] Arce, A. , A. Estirado , M. Ordobas , et al. 2013. “Re‐Emergence of Leishmaniasis in Spain: Community Outbreak in Madrid, Spain, 2009 to 2012.” Euro Surveillance 18, no. 30: 20546. 10.2807/1560-7917.es2013.18.30.20546. 23929177

[zph70086-bib-0007] Ayhan, N. , B. Alten , V. Ivovic , et al. 2017. “Cocirculation of Two Lineages of Toscana Virus in Croatia.” Frontiers in Public Health 5: 336. 10.3389/fpubh.2017.00336. PMC5732939 29312917

[zph70086-bib-0008] Ayhan, N. , and R. N. Charrel . 2020. “An Update on Toscana Virus Distribution, Genetics, Medical and Diagnostic Aspects.” Clinical Microbiology and Infection 26, no. 8: 1017–1023. 10.1016/j.cmi.2019.12.015. 31904562

[zph70086-bib-0009] Ayhan, N. , C. Eldin , and R. Charrel . 2025. “Toscana Virus: A Comprehensive Review of 1381 Cases Showing an Emerging Threat in the Mediterranean Regions.” Journal of Infection 90, no. 2: 106415. 10.1016/j.jinf.2025.106415. 39828129

[zph70086-bib-0010] Ayhan, N. , J. Prudhomme , L. Laroche , A. L. Bañuls , and R. N. Charrel . 2020. “Broader Geographical Distribution of Toscana Virus in the Mediterranean Region Suggests the Existence of Larger Varieties of Sand Fly Vectors.” Microorganisms 8, no. 1: 114. 10.3390/microorganisms8010114. PMC7022675 31947561

[zph70086-bib-0011] Bakhshandeh, B. , Z. Jahanafrooz , A. Abbasi , et al. 2021. “Mutations in SARS‐CoV‐2; Consequences in Structure, Function, and Pathogenicity of the Virus.” Microbial Pathogenesis 154: 104831. 10.1016/j.micpath.2021.104831. PMC7955574 33727169

[zph70086-bib-0012] Barbero‐Moyano, J. , M. Gonzálvez , D. Bravo‐Barriga , et al. 2025. “Epidemiological Landscape in a Mediterranean Hotspot of Human Leishmaniosis in Spain Under a One Health Approach.” Pathogens and Global Health 119, no. 3–4: 122–133. 10.1080/20477724.2025.2480083. PMC12086916 40122138

[zph70086-bib-0013] Barroso, P. , S. Zanet , and E. Ferroglio . 2024. “Meteorological, Demographic, and Environmental Risk Factors for *Leishmania infantum* in Wildlife.” Research in Veterinary Science 173: 105288. 10.1016/j.rvsc.2024.105288. 38718544

[zph70086-bib-0014] Bian, G. , D. Joshi , Y. Dong , et al. 2013. “ *Wolbachia* Invades *Anopheles stephensi* Populations and Induces Refractoriness to *Plasmodium* Infection.” Science 340, no. 6133: 748–751. 10.1126/science.1236192. 23661760

[zph70086-bib-0015] Calzolari, M. , G. Romeo , E. Callegari , et al. 2021. “Co‐Circulation of Phleboviruses and *Leishmania* Parasites in Sand Flies From a Single Site in Italy Monitored Between 2017 and 2020.” Viruses 13, no. 8: 1660. 10.3390/v13081660. PMC8402820 34452524

[zph70086-bib-0016] Charrel, R. N. , A. Izri , S. Temmam , X. de Lamballerie , and P. Parola . 2006. “Toscana Virus RNA in *Sergentomyia minuta* Files.” Emerging Infectious Diseases 12, no. 8: 1299–1300. 10.3201/eid1708.060345. PMC3291233 16972358

[zph70086-bib-0017] Charrel, R. N. , A. Izri , S. Temmam , et al. 2007. “Cocirculation of 2 Genotypes of Toscana Virus, Southeastern France.” Emerging Infectious Diseases 13, no. 3: 465–468. 10.3201/eid1303.061086. PMC2725915 17552102

[zph70086-bib-0018] Charrel, R. N. , G. Moureau , S. Temmam , et al. 2009. “Massilia Virus, a Novel Phlebovirus (Bunyaviridae) Isolated From Sandflies in the Mediterranean.” Vector‐Borne and Zoonotic Diseases 9, no. 5: 519–530. 10.1089/vbz.2008.0131. PMC2976643 19055373

[zph70086-bib-0019] Collao, X. , G. Palacios , F. de Ory , et al. 2010. “Granada Virus: A Natural Phlebovirus Reassortant of the Sandfly Fever Naples Serocomplex With Low Seroprevalence in Humans.” American Journal of Tropical Medicine and Hygiene 83, no. 4: 760–765. 10.4269/ajtmh.2010.09-0697. PMC2946739 20889862

[zph70086-bib-0020] da Silva Gonçalves, D. , I. Iturbe‐Ormaetxe , A. Martins‐da‐Silva , et al. 2019. “ *Wolbachia* Introduction Into *Lutzomyia longipalpis* (Diptera: Psychodidae) Cell Lines and Its Effects on Immune‐Related Gene Expression and Interaction With *Leishmania infantum* .” Parasites & Vectors 12, no. 1: 33. 10.1186/s13071-018-3227-4. PMC6332621 30646951

[zph70086-bib-0021] Davó, L. , L. Herrero , M. P. Sánchez‐Seco , et al. 2020. “Real‐Time RT‐PCR Assay to Detect Granada Virus and the Related Massilia and Arrabida Phleboviruses.” Parasites & Vectors 13, no. 1: 270. 10.1186/s13071-020-04110-5. PMC7257231 32471505

[zph70086-bib-0022] Ergunay, K. , O. E. Kasap , S. Orsten , et al. 2014. “Phlebovirus and *Leishmania* Detection in Sandflies From Eastern Thrace and Northern Cyprus.” Parasites & Vectors 7: 575. 10.1186/s13071-014-0575-6. PMC4269954 25499083

[zph70086-bib-0023] Es‐sette, N. , M. Ajaoud , L. Anga , F. Mellouki , and M. Lemrani . 2015. “Toscana Virus Isolated From Sandflies, Morocco.” Parasites & Vectors 8: 205. 10.1186/s13071-015-0826-1. PMC4415314 25886511

[zph70086-bib-0024] Es‐Sette, N. , M. Ajaoud , L. Bichaud , et al. 2014. “ *Phlebotomus sergenti* a Common Vector of *Leishmania tropica* and Toscana Virus in Morocco.” Journal of Vector Borne Diseases 51, no. 2: 86–90. 24947214

[zph70086-bib-0025] Es‐Sette, N. , J. Nourlil , S. Hamdi , F. Mellouki , and M. Lemrani . 2012. “First Detection of Toscana Virus RNA From Sand Flies in the Genus *Phlebotomus* (Diptera: Phlebotomidae) Naturally Infected in Morocco.” Journal of Medical Entomology 49, no. 6: 1507–1509. 10.1603/me12042. 23270183

[zph70086-bib-0026] Faucher, B. , L. Bichaud , R. Charrel , et al. 2014. “Presence of Sandflies Infected With *Leishmania infantum* and Massilia Virus in the Marseille Urban Area.” Clinical Microbiology and Infection 20, no. 5: O340–O343. 10.1111/1469-0691.12404. 24107240

[zph70086-bib-0027] Fernández‐Arévalo, A. , E. González , C. Ballart , et al. 2024. “Typing of *Leishmania* Isolates From Vectors and Leporids of the Madrid (Spain) Outbreak.” Parasitology 151, no. 2: 213–219. 10.1017/S0031182023001336. PMC10941034 38105582

[zph70086-bib-0028] García San Miguel, L. , M. J. Sierra , A. Vazquez , et al. 2021. “Phlebovirus‐Associated Diseases Transmitted by Phlebotominae in Spain: Are We at Risk?” Enfermedades Infecciosas y Microbiología Clínica (English ed.) 39, no. 7: 345–351. 10.1016/j.eimce.2021.05.001. 34353512

[zph70086-bib-0029] Gijón‐Robles, P. , N. Abattouy , G. Merino‐Espinosa , et al. 2018. “Risk Factors for the Expansion of Cutaneous Leishmaniasis by *Leishmania tropica*: Possible Implications for Control Programmes.” Transboundary and Emerging Diseases 65, no. 6: 1615–1626. 10.1111/tbed.12914. 29806200

[zph70086-bib-0030] Harris, E. K. , V. Balaraman , C. C. Keating , et al. 2025. “Co‐Infection of *Culex tarsalis* Mosquitoes With Rift Valley Fever Phlebovirus Strains Results in Efficient Viral Reassortment.” Viruses 17, no. 1: 88. 10.3390/v17010088. PMC11768849 39861876

[zph70086-bib-0031] Hong, X. G. , M. Q. Zhang , F. Tang , et al. 2024. “Epidemiology and Ecology of Toscana Virus Infection and Its Global Risk Distribution.” Viruses 17, no. 1: 15. 10.3390/v17010015. PMC11768567 39861803

[zph70086-bib-0032] Huemer, H. , J. Prudhomme , F. Amaro , et al. 2017. “Practical Guidelines for Studies on Sandfly‐Borne Phleboviruses: Part II: Important Points to Consider for Fieldwork and Subsequent Virological Screening.” Vector Borne and Zoonotic Diseases 17, no. 1: 81–90. 10.1089/vbz.2016.1965. 28055572

[zph70086-bib-0033] Jancarova, M. , N. Polanska , A. Thiesson , et al. 2025. “Susceptibility of Diverse Sand Fly Species to Toscana Virus.” PLoS Neglected Tropical Diseases 19, no. 5: e0013031. 10.1371/journal.pntd.0013031. PMC12047804 40315233

[zph70086-bib-0034] Keskek Turk, Y. , K. Ergunay , A. Kohl , J. Hughes , and C. S. McKimmie . 2024. “Toscana Virus—An Emerging Mediterranean Arbovirus Transmitted by Sand Flies.” Journal of General Virology 105, no. 11: 002045. 10.1099/jgv.0.002045. PMC11542635 39508743

[zph70086-bib-0035] Khongpetch, J. , G. Moirano , A. Grisendi , et al. 2025. “Seasonal Trends of Sand Fly Abundance and *Leishmania* Infection: The Case of Bologna Province, Italy (2016–2023).” One Health 20: 101050. 10.1016/j.onehlt.2025.101050. PMC12434504 40959799

[zph70086-bib-0036] Laroche, L. , A. L. Bañuls , R. Charrel , A. Fontaine , N. Ayhan , and J. Prudhomme . 2024. “Sand Flies and Toscana Virus: Intra‐Vector Infection Dynamics and Impact on *Phlebotomus perniciosus* Life‐History Traits.” PLoS Neglected Tropical Diseases 18, no. 9: e0012509. 10.1371/journal.pntd.0012509. PMC11458028 39321202

[zph70086-bib-0037] Latrofa, M. S. , I. Varotto‐Boccazzi , V. N. Louzada‐Flores , et al. 2023. “Interaction Between *Wolbachia pipientis* and *Leishmania infantum* in Heartworm Infected Dogs.” Parasites & Vectors 16, no. 1: 77. 10.1186/s13071-023-05662-y. PMC9972713 36850014

[zph70086-bib-0038] Martinez, J. G. , S. G. García , S. Walter , et al. 2022. “Seroprevalence Against Toscana Virus in Spain: The Case of the Autonomous Community of Madrid.” Journal of Vector Borne Diseases 59, no. 2: 172–177. 10.4103/0972-9062.335771. 36124483

[zph70086-bib-0039] Martín‐Sánchez, J. , V. Díaz‐Sáez , F. Morillas‐Márquez , et al. 2024. “Wild Rabbits Are *Leishmania infantum* Reservoirs in Southeastern Spain.” Zoonoses and Public Health 71, no. 5: 584–590. 10.1111/zph.13139. 38693773

[zph70086-bib-0040] Martín‐Sánchez, J. , I. Pedrosa‐Corral , A. Torres‐Llamas , et al. 2026. “Entomological Surveillance of Dwellings and Risk Factors for Autochthonous Cases of Human Leishmaniasis in a European Mediterranean Hotspot.” Acta Tropica 278: 108086. 10.1016/j.actatropica.2026.108086. 41956275

[zph70086-bib-0041] Martín‐Sánchez, J. , J. Rodríguez‐Granger , F. Morillas‐Márquez , et al. 2020. “Leishmaniasis due to *Leishmania infantum*: Integration of Human, Animal and Environmental Data Through a One Health Approach.” Transboundary and Emerging Diseases 67, no. 6: 2423–2434. 10.1111/tbed.13580. 32304278

[zph70086-bib-0042] Molina, R. , M. I. Jiménez , I. Cruz , et al. 2012. “The Hare ( *Lepus granatensis* ) as Potential Sylvatic Reservoir of *Leishmania infantum* in Spain.” Veterinary Parasitology 190, no. 1–2: 268–271. 10.1016/j.vetpar.2012.05.006. 22677135

[zph70086-bib-0043] Morales‐Yuste, M. , J. Martín‐Sánchez , and V. Corpas‐Lopez . 2022. “Canine Leishmaniasis: Update on Epidemiology, Diagnosis, Treatment, and Prevention.” Veterinary Sciences 9, no. 8: 387. 10.3390/vetsci9080387. PMC9416075 36006301

[zph70086-bib-0044] Moreira, L. A. , I. Iturbe‐Ormaetxe , J. A. Jeffery , et al. 2009. “A *Wolbachia* Symbiont in *Aedes aegypti* Limits Infection With Dengue, Chikungunya, and *Plasmodium* .” Cell 139, no. 7: 1268–1278. 10.1016/j.cell.2009.11.042. 20064373

[zph70086-bib-0045] Moriconi, M. , G. Rugna , M. Calzolari , et al. 2017. “Phlebotomine Sand Fly‐Borne Pathogens in the Mediterranean Basin: Human Leishmaniasis and Phlebovirus Infections.” PLoS Neglected Tropical Diseases 11, no. 8: e0005660. 10.1371/journal.pntd.0005660. PMC5552025 28796786

[zph70086-bib-0046] Navarro‐Marí, J. M. , C. Gómez‐Camarasa , M. Pérez‐Ruiz , S. Sanbonmatsu‐Gámez , I. Pedrosa‐Corral , and M. Jiménez‐Valera . 2013. “Clinic‐Epidemiologic Study of Human Infection by Granada Virus, a New Phlebovirus Within the Sandfly Fever Naples Serocomplex.” American Journal of Tropical Medicine and Hygiene 88, no. 5: 1003–1006. 10.4269/ajtmh.12-0732. PMC3752739 23419365

[zph70086-bib-0047] Navarro‐Marí, J. M. , B. Palop‐Borrás , M. Pérez‐Ruiz , and S. Sanbonmatsu‐Gámez . 2011. “Serosurvey Study of Toscana Virus in Domestic Animals, Granada, Spain.” Vector‐Borne and Zoonotic Diseases 11, no. 5: 583–587. 10.1089/vbz.2010.0065. 20925529

[zph70086-bib-0048] Peyrefitte, C. N. , M. Grandadam , M. Bessaud , et al. 2013. “Diversity of *Phlebotomus perniciosus* in Provence, Southeastern France: Detection of Two Putative New Phlebovirus Sequences.” Vector Borne and Zoonotic Diseases 13, no. 9: 630–636. 10.1089/vbz.2012.1169. 23705585

[zph70086-bib-0049] Pimentel, A. C. , C. S. Cesar , A. H. B. Martins , M. Martins , and R. Cogni . 2025. “ *Wolbachia* Offers Protection Against Two Common Natural Viruses of *Drosophila* .” Microbial Ecology 88, no. 1: 24. 10.1007/s00248-025-02518-z. PMC11982076 40202691

[zph70086-bib-0050] Polat, C. , N. Ayhan , K. Ergünay , and R. N. Charrel . 2024. “Comprehensive Evaluation of Nucleic Acid Amplification Methods Widely Used for Generic Detection of Sandfly‐Borne Phleboviruses.” Microbiology Spectrum 12, no. 4: e0342823. 10.1128/spectrum.03428-23. PMC10986501 38456695

[zph70086-bib-0051] Rath, C. T. , L. C. Schnellrath , C. R. Damaso , et al. 2019. “Amazonian *Phlebovirus* (*Bunyaviridae*) Potentiates the Infection of *Leishmania* (*Leishmania*) *amazonensis*: Role of the PKR/IFN1/IL‐10 Axis.” PLoS Neglected Tropical Diseases 13, no. 6: e0007500. 10.1371/journal.pntd.0007500. PMC6602282 31216268

[zph70086-bib-0052] Remoli, M. E. , M. Jiménez , C. Fortuna , et al. 2016. “Phleboviruses Detection in *Phlebotomus perniciosus* From a Human Leishmaniasis Focus in South‐West Madrid Region, Spain.” Parasites & Vectors 9: 205. 10.1186/s13071-016-1488-3. PMC4831143 27075742

[zph70086-bib-0053] Rivas Martínez, S. , J. M. Gandullo , R. Serrada , J. L. Allué Andrade , J. L. Montero de Burgos , and J. L. González Rebollar . 1987. Memoria Del Mapa de Series de Vegetación de España, 31–42. Ministerio de Agricultura, Pesca y Alimentación, ICONA.

[zph70086-bib-0054] Rocha, R. , E. Kurum , N. Ayhan , R. Charrel , and C. Maia . 2025. “Seroprevalence of Toscana Virus in Blood Donors in Mainland Portugal.” Parasites & Vectors 18, no. 1: 82. 10.1186/s13071-025-06726-x. PMC11874794 40033431

[zph70086-bib-0055] Rosário, A. A. D. , A. G. Dias‐Lima , S. M. Lambert , B. M. P. D. S. Souza , and F. Bravo . 2022. “Identification and Molecular Characterization of *Wolbachia* Strains and Natural Infection for *Leishmania* sp. in Neotropical Phlebotominae (Diptera: Psychodidae) Species, Leishmaniasis Vectors.” Acta Tropica 235: 106624. 10.1016/j.actatropica.2022.106624. 35914568

[zph70086-bib-0056] Rossi, M. , P. Castiglioni , M. A. Hartley , et al. 2017. “Type I Interferons Induced by Endogenous or Exogenous Viral Infections Promote Metastasis and Relapse of Leishmaniasis.” Proceedings of the National Academy of Sciences of the United States of America 114, no. 19: 4987–4992. 10.1073/pnas.1621447114. PMC5441690 28439019

[zph70086-bib-0057] Roy, M. , S. Lacôte , S. Desloire , et al. 2025. “Two Strains of Toscana Virus Show Different Virulence and Replication Capacity in Mice and Cell Culture Models.” Virulence 16, no. 1: 2535470. 10.1080/21505594.2025.2535470. PMC12296115 40693587

[zph70086-bib-0058] Sáez, V. D. , F. Morillas‐Márquez , G. Merino‐Espinosa , et al. 2018. “ *Phlebotomus langeroni* Nitzulescu (Diptera, Psychodidae) a New Vector for *Leishmania infantum* in Europe.” Parasitology Research 117, no. 4: 1105–1113. 10.1007/s00436-018-5788-8. 29404748

[zph70086-bib-0059] Sanbonmatsu‐Gámez, S. , M. Pérez‐Ruiz , X. Collao , et al. 2005. “Toscana Virus in Spain.” Emerging Infectious Diseases 11, no. 11: 1701–1707. 10.3201/eid1111.050851. PMC3367369 16318721

[zph70086-bib-0060] Sánchez‐Seco, M. P. , J. M. Echevarría , L. Hernández , D. Estévez , J. M. Navarro‐Marí , and A. Tenorio . 2003. “Detection and Identification of Toscana and Other Phleboviruses by RT‐Nested‐PCR Assays With Degenerated Primers.” Journal of Medical Virology 71, no. 1: 140–149. 10.1002/jmv.10465. 12858420

[zph70086-bib-0061] Stejskalova, M. , M. Jancarova , K. Pruzinova , et al. 2025. “Impact of *Asaia* Bacteria on *Leishmania major* Development in Sand Flies: Implications for Vector Control Strategies.” Parasites & Vectors 18, no. 1: 424. 10.1186/s13071-025-07075-5. PMC12542282 41126347

[zph70086-bib-0062] Stejskalova, M. , N. Polanska , S. Desloire , M. Ratinier , P. Volf , and M. Jancarova . 2026. “Negative Interactions Between Toscana Virus and *Leishmania infantum* Limit Coinfection in Sand Flies.” Parasites & Vectors 19, no. 1: 121. 10.1186/s13071-025-07237-5. PMC12997898 41680948

[zph70086-bib-0063] Teixeira, L. , A. Ferreira , and M. Ashburner . 2008. “The Bacterial Symbiont *Wolbachia* Induces Resistance to RNA Viral Infections in *Drosophila melanogaster* .” PLoS Biology 6, no. 12: e2. 10.1371/journal.pbio.1000002. PMC2605931 19222304

[zph70086-bib-0064] Thiesson, A. , M. P. Confort , S. Desloire , A. Kohl , F. Arnaud , and M. Ratinier . 2025. “Genetic Diversity of Toscana Virus Glycoproteins Affects the Kinetics of Virus Entry and the Infectivity of Newly Produced Virions.” Npj Viruses 3, no. 1: 28. 10.1038/s44298-025-00113-0. PMC12000347 40295709

[zph70086-bib-0065] Torres‐Llamas, A. , V. Díaz‐Sáez , M. Morales‐Yuste , et al. 2025. “Assessing *Wolbachia* Circulation in Wild Populations of Phlebotomine Sand Flies From Spain and Morocco: Implications for Control of Leishmaniasis.” Parasites & Vectors 18, no. 1: 155. 10.1186/s13071-025-06771-6. PMC12032678 40287743

[zph70086-bib-0066] Vadmal, G. M. , C. K. Glidden , B. A. Han , B. M. Carvalho , A. A. Castellanos , and E. A. Mordecai . 2023. “Data‐Driven Predictions of Potential *Leishmania* Vectors in the Americas.” PLoS Neglected Tropical Diseases 17, no. 2: e0010749. 10.1371/journal.pntd.0010749. PMC9983874 36809249

[zph70086-bib-0067] Varotto‐Boccazzi, I. , S. Epis , I. Arnoldi , et al. 2020. “Boosting Immunity to Treat Parasitic Infections: Asaia Bacteria Expressing a Protein From *Wolbachia* Determine M1 Macrophage Activation and Killing of *Leishmania* Protozoans.” Pharmacological Research 161: 105288. 10.1016/j.phrs.2020.105288. 33160070

[zph70086-bib-0068] Vaselek, S. 2021. “Systematic Review: Re‐Emergence of Human Leishmaniasis in the Balkans.” Tropical Medicine & International Health 26, no. 10: 1189–1199. 10.1111/tmi.13653. 34265146

[zph70086-bib-0069] Wang, D. , A. R. Hof , K. D. Matson , F. van Langevelde , and CLIMOS data providers . 2025. “Understanding and Predicting the Geographic Distributions of Phlebotomine Sand Flies in and Around Europe.” Climatic Change 178, no. 11: 205. 10.1007/s10584-025-04009-z. PMC12589297 41209429

[zph70086-bib-0070] Weidmann, M. , M. P. Sanchez‐Seco , A. A. Sall , et al. 2008. “Rapid Detection of Important Human Pathogenic Phleboviruses.” Journal of Clinical Virology 41, no. 2: 138–142. 10.1016/j.jcv.2007.10.001. 18006376

[zph70086-bib-0071] Wilson, A. L. , O. Courtenay , L. A. Kelly‐Hope , et al. 2020. “The Importance of Vector Control for the Control and Elimination of Vector‐Borne Diseases.” PLoS Neglected Tropical Diseases 14, no. 1: e0007831. 10.1371/journal.pntd.0007831. PMC6964823 31945061

[zph70086-bib-0072] Wong, M. L. , J. W. K. Liew , W. K. Wong , et al. 2020. “Natural *Wolbachia* Infection in Field‐Collected *Anopheles* and Other Mosquito Species From Malaysia.” Parasites & Vectors 13, no. 1: 414. 10.1186/s13071-020-04277-x. PMC7425011 32787974

[zph70086-bib-0073] Xhekaj, B. , E. Kurum , J. Stefanovska , et al. 2025. “Neutralization‐Based Seroprevalence of Toscana Virus and Sandfly Fever Sicilian Virus in Dogs in the Republic of Kosovo.” Parasites & Vectors 18, no. 1: 48. 10.1186/s13071-025-06681-7. PMC11812177 39930491

[zph70086-bib-0074] Yilmaz, A. , and O. E. Kasap . 2025. “Prevalence of *Wolbachia* in Natural Sand Fly (Diptera: Psychodidae) Populations From Türkiye and Its Potential Role in Mitochondrial Divergence.” Parasites & Vectors 19, no. 1: 16. 10.1186/s13071-025-07157-4. PMC12797604 41345699

[zph70086-bib-0075] Zhou, W. , F. Rousset , and S. O'Neil . 1998. “Phylogeny and PCR‐Based Classification of *Wolbachia* Strains Using Wsp Gene Sequences.” Proceedings. Biological sciences 265, no. 1395: 509–515. 10.1098/rspb.1998.0324. PMC1688917 9569669

