# Supplementary material for: Natural Infection by Toscana Virus and Other Phleboviruses in Wild Sand Flies From Spain: Prevalence, Genetic Characterization and Tripartite Interactions With Leishmania infantum and Wolbachia
Source: Zoonoses Public Health. 2026 Aug 20;73(7):655–69. doi: 10.1111/zph.70086 (PMC13633447; doi:10.1111/zph.70086)
Supplement: Supplementary file 1 [file ZPH-73-655-s001.docx]

**Supplementary material**

**Primers used in the L segment**

**PCR**

| NPhlebo1+ | 5’—TGGARGGITTTGTIWSICIICC—3’ | 550-bp |
| --- | --- | --- |
| NPhlebo1– | 5’—AARTTRCTIGWIGCYTTIARIGTIGC—3’ |  |

Briefly, 12.5 µL of bidistilled water, 2.5 µL 10X buffer, 2 µL 50 mM MgCl_2_, 1 µL 10 mM dNTP_s,_ 1 µL 20 mM forward and reverse primers, 1 µL 1.25 U Taq polymerase, and 4 µL cDNA were added to the reaction tube. The amplification conditions were: 2 min denaturation at 94 °C followed by 40 cycles of denaturation at 94 °C for 30 s, annealing at 45 °C for 1 min, and extension at 72 °C for 40 s, with a final extension at 72 °C for 5 min.

**Nested-PCR**

| NPhlebo2+ | 5’— WTICCIAAICCIYMSAARATG—3’ | 244-bp |
| --- | --- | --- |
| NPhlebo2– | 5’— TCYTCYTTRTTYTTRARRTARCC—3’ |  |

Briefly, 12.5 µL of bidistilled water, 2.5 µL 10X buffer, 2 µL 50 mM MgCl_2_, 1 µL 10 mM dNTP_s,_ 1 µL 20 mM forward and reverse primers, 1 µL 1.25 U Taq polymerase, and 4 µL cDNA were added to the reaction tube. The amplification conditions were: 2 min denaturation at 94 °C followed by 40 cycles of denaturation at 94 °C for 30 s, annealing at 45 °C for 1 min, and extension at 72 °C for 40 s, with a final extension at 72 °C for 5 min.

| NPhlebo2+ | 5’— WTICCIAAICCIYMSAARATG—3’ | 126-bp |
| --- | --- | --- |
| Atos2– | 5’— TCYTCYTTRTTYTTRARRTARCC—3’ |  |

Briefly, 13 µL of bidistilled water, 2.5 µL 10X buffer, 1.5 µL 50 mM MgCl_2_, 1 µL 10 mM dNTP_s,_ 1 µL 20 mM forward and reverse primers, 1 µL 1.25 U Taq polymerase, and 4 µL cDNA were added to the reaction tube. The amplification conditions were: 2 min denaturation at 94 °C followed by 40 cycles of denaturation at 94 °C for 30 s, annealing at 45 °C for 1 min, and extension at 72 °C for 40 s, with a final extension at 72 °C for 5 min.

**Primers used in the S segment**

**PCR**

| TosN123 | 5’— GAGTTTGCTTACCAAGGGTTTG—3’ | 708-bp |
| --- | --- | --- |
| TosN829 | 5’— AATCCTAATTCCCCTAACCCCC—3’ |  |

Briefly, 13.5 µL of bidistilled water, 2.5 µL 10X buffer, 1 µL 50 mM MgCl_2_, 1 µL 10 mM dNTP_s,_ 1 µL 20 mM forward and reverse primers, 1 µL 1.25 U Taq polymerase, and 4 µL cDNA were added to the reaction tube. The amplification conditions were: 2 min denaturation at 94 °C followed by 40 cycles of denaturation at 94 °C for 30 s, annealing at 50 °C for 30 s, and extension at 72 °C for 1 min.

**Nested-PCR**

| TosN234 | 5’— AACCTTGTCAGGGGNAACAAGCC—3’ | 560-bp |
| --- | --- | --- |
| TosN794 | 5’— GCCAACCTTGGCGCGATACTTC—3’ |  |

Briefly, 13.5 µL of bidistilled water, 2.5 µL 10X buffer, 1 µL 50 mM MgCl_2_, 1 µL 10 mM dNTP_s,_ 1 µL 20 mM forward and reverse primers, 1 µL 1.25 U Taq polymerase, and 4 µL cDNA were added to the reaction tube. The amplification conditions were: 2 min denaturation at 94 °C followed by 40 cycles of denaturation at 94 °C for 30 s, annealing at 56 °C for 30 s, and extension at 72 °C for 40 s.
